# Wetting-Induced Polyelectrolyte Pore Bridging

**DOI:** 10.3390/membranes11090671

**Published:** 2021-08-31

**Authors:** Anna Kalde, Johannes Kamp, Elizaveta Evdochenko, John Linkhorst, Matthias Wessling

**Affiliations:** 1 DWI-Leibniz—Institute for Interactive Materials, Forckenbeckstrasse 50, 52074 Aachen, Germany; anna.kalde@avt.rwth-aachen.de; 2 Chemical Process Engineering, RWTH Aachen University, Forckenbeckstrasse 51, 52074 Aachen, Germany; Johannes.Kamp@avt.rwth-aachen.de (J.K.); elizaveta.evdochenko@avt.rwth-aachen.de (E.E.); john.linkhorst@avt.rwth-aachen.de (J.L.)

**Keywords:** polyelectrolyte multilayer membrane, layer by layer, nanofiltration, polyelectrolyte complexation

## Abstract

Active layers of ion separation membranes often consist of charged layers that retain ions based on electrostatic repulsion. Conventional fabrication of these layers, such as polyelectrolyte deposition, can in some cases lead to excess coating to prevent defects in the active layer. This excess deposition increases the overall membrane transport resistance. The study at hand presents a manufacturing procedure for controlled polyelectrolyte complexation in and on porous supports by support wetting control. Pre-wetting of the microfiltration membrane support, or even supports with larger pore sizes, leads to ternary phase boundaries of the support, the coating solution, and the pre-wetting agent. At these phase boundaries, polyelectrolytes can be complexated to form partially freestanding selective structures bridging the pores. This polyelectrolyte complex formation control allows the production of membranes with evenly distributed polyelectrolyte layers, providing (1) fewer coating steps needed for defect-free active layers, (2) larger support diameters that can be bridged, and (3) a precise position control of the formed polyelectrolyte multilayers. We further analyze the formed structures regarding their position, composition, and diffusion dialysis performance.

## 1. Introduction

Water scarcity and climate change drive the need for sustainable industrial processes [1]. To prevent pollution via produced water, often membrane filtration processes are crucial to meet purification requirements [2]. Nanofiltration is a powerful tool to purify highly polluted water by removing small organic molecules and ions. For ion removal, nanofiltration membranes typically contain fixed charged groups which repel mostly polyvalent ions. Obtaining fixed charged groups in a nanofiltration membrane can be achieved with different methods, such as interfacial polymerization, or the coating of an ion-selective layer on a porous support. One prominent approach to form such layers uses polyelectrolytes that are, for instance, assembled on a microfiltration membrane [3,4]. Here, the complexation of opposingly charged polyelectrolytes is used to form solid layers for ion removal. Various applications use different polyelectrolyte complex formation methods [5]. Examples are spraying, photolithography, electrochemical deposition, spin-coating, and microcontact printing [6,7,8]. Further, also convective layer by layer assembly [9], and direct spinning [10] have been presented in the literature. Often, a layer by layer (LbL) assembly via dip coating is used to fabricate a precisely tailored polyelectrolyte coating [2,11,12,13,14].

All coating methods presented in the literature show increasing membrane selectivity and transport resistance across the membrane with increasing amounts of polyelectroyltes adsorbed and complexated on the support. To achieve defect-free active layers, multiple coating steps are mostly necessary because small defects in the first coating layers need to be covered by further coating layers. In turn, this increases the transport resistance for water or other liquids to pass the membrane. While membrane selectivity should be as high as possible, low transport resistances are needed for an economical process. Therefore, reducing polyelectrolyte coating thickness while maintaining defect-free layers is a crucial factor in improving the energy efficiency of nanofiltration membranes. Hence, the deposition behavior of polyelectrolytes and polyelectrolyte complex formation often is in focus in the literature [15,16,17,18]. In LbL polyelectrolyte assembly, this trade-off between selectivity and transport resistance is a natural dilemma because of the inhomogeneous microfiltration support pore size and local intrusion of the polyelectrolytes. Small pores of the support membrane are easily coated within a few coating steps, whereas the bigger pores need additional coating steps to be sealed with a selective layer [19]. With these additional coating steps, the smaller pores’ coating unnecessarily thickens, which again increases transport resistance. Since various polyelectrolyte layer compositions have been studied and highly ion-selective combinations and coating parameters have been presented in the literature [4,9,20,21], the study at hand focuses on the control of polyelectrolyte complex thickness and position that can be applied to tailored polyelectrolyte combinations in future work.

The production of thin freestanding, or reinforced polyelectrolyte layers is widely studied in literature [6,13,22,23,24,25,26]. Reported manufacturing methods involve salt dilution induced phase separation [27,28], sacrificial layers [22,29,30,31,32,33,34,35], deposition at liquid–liquid interfaces [13,36], deposition at gas–liquid interfaces [23,37,38,39], and deposition by solvent evaporation [40,41]. Mallwitz et al. [42] proposed a method to span PAA/PAH complexes over electron microscopy grids with hole sizes of 100 μm. However, these freestanding structures are not applicable for large scale production for water applications as they are not mechanically stable, the production procedures are complex, or require sophisticated process control.

In general, polyelectrolyte deposition in LbL technology is based on the adsorption and complexation of a polyelectrolyte on the opposingly charged polyelectrolyte layer from a previous coating step. Conventionally, the intrinsic charge of a porous substrate gives rise to the adsorption of polyelectrolytes for the first coating steps. Conventional layer by layer technology usually thoroughly wets the macroporous support with a coating solution during the membrane fabrication process. Consequently, the polyelectrolyte adsorption takes place both at the support surface and inside the porous support [43]. Depending on the support geometry, different topography of selective layers arise, leading to different membrane performances. Especially in general nanofiltration material characterization, this is undesirable since the experimental results are dependent on uncontrolled parameters.

Further, polyelectrolytes tend to remain near fluid–fluid interfaces due to their often amphiphilic nature. To create such a fluid–fluid interface for polyelectrolyte enrichment in a first coating step, we induce a fluid–fluid interface inside the pores by controlling the wetting state of the support structure. The wetting state can be controlled via back-pressure control of air, or immersion of a pre-wetting agent. To obtain a stable interface, pore-filling fluid must effectively prevent the coating solution from intruding the pore throat. In turn, the applied pressure on the pore-filling fluid may not exceed the breakthrough pressure $p_{B}$ of the pore-filling fluid. In this case, a constant flow of air or pre-wetting fluid would prohibit the formation of any pore-spanning layer. Hence, no net flow of air or pre-wetting fluid across the membrane may occur.

The breakthrough pressure can be calculated according to the Young-Laplace equation as denoted in Equation (1), where γ denotes the surface tension of the fluid, θ is the respective contact angle, and *d* is the pore diameter. In a porous material with heterogeneous pore sizes, a breakthrough will always occur first in the largest pores.

(1) $$p_{b}=\frac{\gamma \cos\theta }{d}$$

By controlling the location of polyelectrolyte deposion at a pore scale, the study at hand presents a straightforward approach to even out the number of coating steps needed. The formation of pore-spanning polyelectrolyte complexes leads to a full coverage of the support structure at a drastically reduced number of coating steps compared to pure adsorption at the pore walls. As the pre-wetting of the support structure is a well-controllable process, it does not negatively affect the coating process itself, while enhancing the obtained results regarding large pores.

The wetting control of the support induces the built-up of pore-spanning polyelectrolyte layers within few coating steps and uniform polyelectrolyte deposition over the pores. Hence, this method might be a possible new way to decrease the transport resistance and use less coating steps for membrane production. The controllable membrane thickness throughout the support might be beneficial for future testing of novel membrane materials or parameter studies on membrane performance, as the influence of the support can be estimated and kept constant for different materials. Further, the method might be useful in membrane production for diffusion-based processes, such as control of ionic strength in biotechnological systems. However, the here presented proof of concept of a novel polyelectrolyte multilayer membrane (PEMM) assembly method focuses on the general feasibility of the production concept. We show that defect-free membranes consisting of well-known materials can be manufactured with few coating steps. Highly controlled PEMM can be generated with only little process control applied. To be suitable for a scale-up to large membrane areas, further optimization regarding application-oriented coating material and mechanical stability is necessary.

## 2. Materials and Methods

### 2.1. Support Structure

Flat isoporous silicon support structures with diameters of 10 mm to 25 mm purchased from SmartMembranes GmbH, Germany, were used for all presented experiments. The isoporous supports had a pore size of 1 μm, a lattice constant of 1.5 μm, and a pore length of 200 μm. The standard deviation in pore diameter was below 5%. The pores were in a trigonal order.

Additionally, coating experiments were repeated with a commercial membrane as heteroporous support with comparable pore size to the used silicon support, presented in Section 3.5. To visually assess the wetting control during polyelectrolyte complexation, we applied the coating on the shell side of a hollow fiber membrane. A commercial Pentair X-Flow ultrafiltration membrane with a nominal molecular weight cut-off of 150–200 kDa was used. This membrane type had a selective microfiltration layer on the lumen side and a wide-open porous topography on its shell. Therefore, we used the commercial membrane’s support structure on the shell side as erratic support for PEMM assembly without any influence from the ultrafiltration layer on the lumen side.

To compare the produced PEMM layer growth to conventionally dip-coated PEMM, we additionally coated silicon wafers with a native oxide surface without chemical pre-treatment. We used the same coating conditions as for the isoporous silicon support structures. The results in PEMM thicknesses are presented in Section 3.2.

### 2.2. Polyelectrolyte Complexation

Poly-diallyl dimethylammonium chloride (PDADMAC, 100 kDa, Sigma-Aldrich Chemie GmbH, Schnelldorf, Germany) was used as polycation, and polystyrene sulfonate (PSS, 70 kDa, Sigma-Aldrich Chemie GmbH, Schnelldorf, Germany) was used as polyanion for active layer fabrication. For the coating solutions, 1 g/L polyelectrolyte and 0.5 M NaCl were used. This combination of polyelectrolytes with the respective counter ion concentration for LbL applications is known to show high adsorption rates of polyelectrolytes, but low salt retentions [44,45].

**Figure 1:**
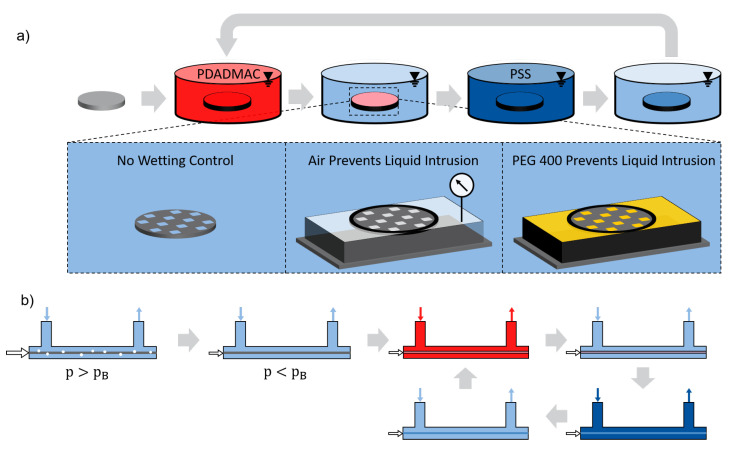
Schematic setup used for polyelectrolyte deposition. (**a**) Static coating of flat isoporous supports. Alternating dip-coating of samples leads to successive polyelectrolyte deposition. Isoporous supports without any periphery, supports sealed to an air-filled compartment with 10 mbar excess pressure, and supports sealed to a compartment filled with PEG400 were dip-coated. (**b**) Dynamic coating of a heterogeneous hollow fiber support. Air flows through the lumen at a pressure level $p>p_{B}$, while the module is filled with water. The gas pressure is reduced until $p<p_{B}$. Polyelectrolyte coating solutions and rinsing solutions are alternatingly pumped through the shell side of the module.

Polyelectrolyte complexation was conducted via an LbL dip-coating procedure with alternatingly contacting the support with polyanion and polycation coating baths, as can be seen in Figure 1. We chose the first polyelectrolyte layer to be cationic since the support naturally has a slightly negative surface charge. The sample dwell time in the coating baths was 15 min. A 20 min washing step in distilled water to remove excess polyelectrolytes was conducted after each coating step. For this washing step, the isoporous silicon supports were inserted in a rinsing bath, whereas the hollow fibers were rinsed with a cross-flow of 15 mL/min over 20 min. For the hollow fiber modules, this corresponds to a turnover of five times the module volume. After PEMM fabrication, samples were stored in water.

### 2.3. Wetting Agents and Wetting Control

Flat isoporous supports are coated in three different wetting configurations, as depicted schematically in Figure 1a. For reference, first, a fully water-wetted support structure was dip-coated without any periphery. Second, isoporous supports were sealed to a sample holder, providing an air-filled compartment at the backside of the support. In this compartment, the gas pressure can be controlled. The support was dip-coated while intrusion of the coating solution into the pores was prevented by the applied air pressure. This way, a gas–liquid interface was generated near the pore throat. Third, the compartment at the support backside was filled with polyethylene glycol with an average molecular weight of 400 Da (PEG400), purchased from Sigma-Aldrich, Germany. The PEG400 was filled in the compartment until it reached the support pore throats at the coating side. Subsequently, the PEG400-filled support was dip-coated with no further pressure control applied. All flat samples were coated horizontally to avoid pressure gradients due to different immersion depths. No chemical pre-treatment was applied to the silicon supports in any other way.

Additionally, isoporous supports with air-filled pores were coated with one bilayer of polyelectrolytes. To test the stability of the initial polyelectrolyte bilayer spanning the pores, the samples were excerpted from the sample holder and coated with another two bilayers without any further wetting control.

Hollow fiber samples were prepared using commercial hollow fiber membranes as support structures. The fibers were assembled in transparent single-membrane modules with 15 cm free membrane length. Modules were designed for cross-flow filtration, comprising inlets and outlets both for lumen and shell side. For the hollow fiber coating, a dead-end configuration was used by closing the lumen outlet. A schematic depiction of the applied coating procedure can be found in Figure 1b. In a first step, a constant air flow was applied through the membrane directed inside out with an excess pressure of 400 mbar. Subsequently, the membrane module was filled with water. Once a steady flow of 15 mL/min was established, the gas pressure was reduced until the gas flow was fully stopped. The residual gas pressure was kept constant for the rest of the coating procedure. To form polyelectrolyte layers, the module’s shell side was repeatedly filled with coating- and rinsing solutions.

### 2.4. Analytical Methods

**Figure 2:**
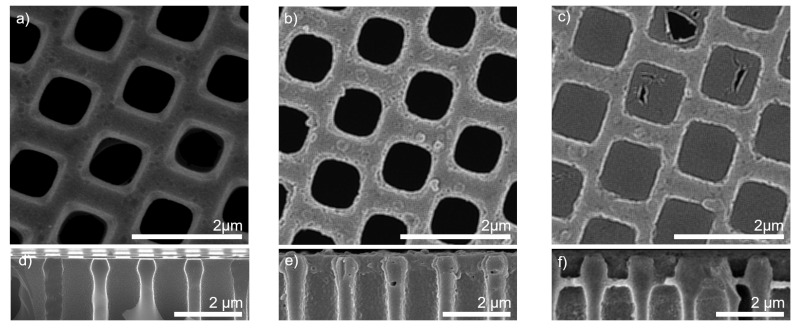
FESEM imaging of isoporous silicon support structures. (**a**) top view and (**d**) cross sectional view of silicon support structure withouth coating; (**b**) top view and (**e**) cross sectional view of three bilayers PDADMAC/PSS on isoporous silicon support with fully water-wetted suppport during polyelectrolyte complexation; (**c**) top view and (**f**) cross sectional view of three bilayers PDADMAC/PSS on silicon support with air-filled support during polyelectrolyte complexation.

**Figure 3:**
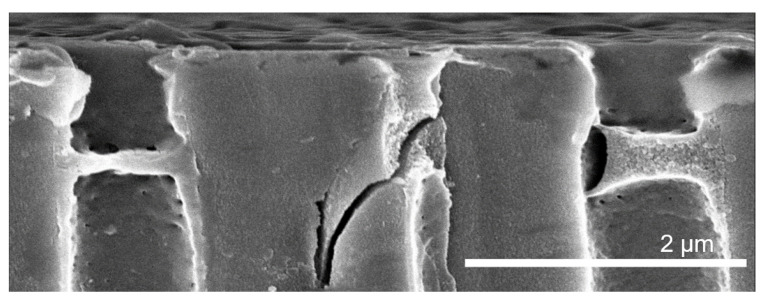
FESEM imaging of porous silicon support cross-section with three bilayers PDADMAC/PSS with air-filled support during polyelectrolyte complexation. Left pore: prominent axial shrinking of polyelectrolytes, right pore: prominent radial shrinking of polyelectrolytes due to wall-detachment.

**Figure 4:**
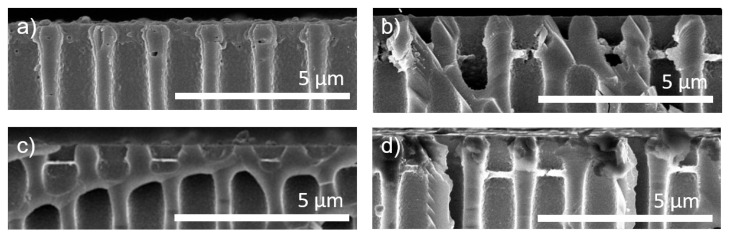
FESEM imaging of porous silicon support cross-sections with three bilayers PDADMAC/PSS with (**a**) fully water-wetted support; (**b**) fully air-filled support; (**c**) air-filled support during the first two coating steps and subsequently no wetting control, and (**d**) PEG400-wetted support during polyelectrolyte complexation.

**Figure 5:**
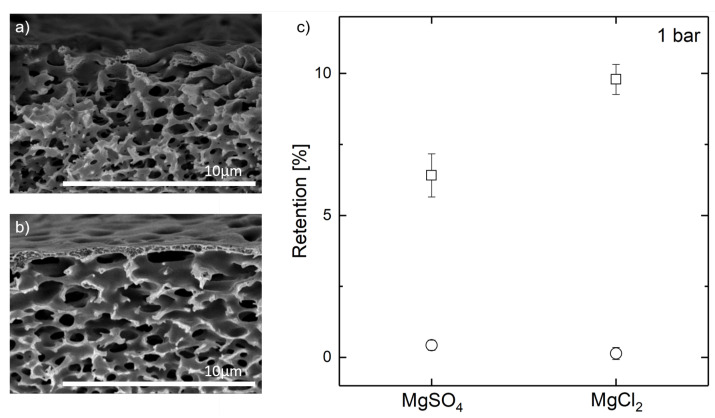
FESEM imaging of a polyethersulphone commercial membrane support structure. (**a**) without polyelectrolyte coating, and (**b**) with three bilayers PDADMAC/PSS with air-filled support during polyelectrolyte complexation. (**c**) Retention of MgCl_2_ and MgSO_4_ at 1 bar TMP of a non-coated membrane (circles) and a hollow fiber coated with five bilayers of PDADMAC/PSS with simultaneous wetting control by air back-pressure (rectangles). Experiments were performed on three membrane modules each.

**Figure 6:**
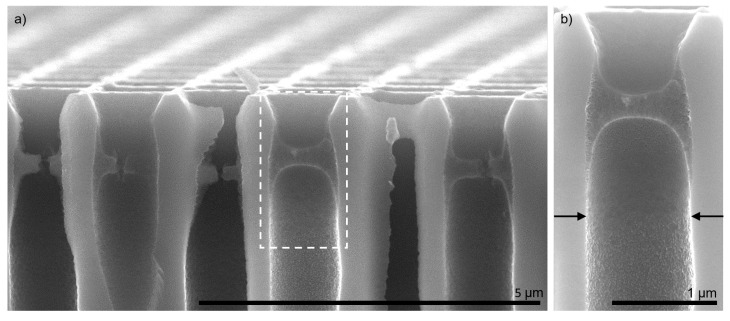
Cross sectional view of three bilayers PDADMAC/PSS on isoporous silicon support with fully air-filled support during polyelectrolyte complexation. Dashed rectangle in (**a**) marks area of magnification for (**b**). Arrows in (**b**) indicate immersion depth of polyelectrolytes into the pore.

**Figure 7:**
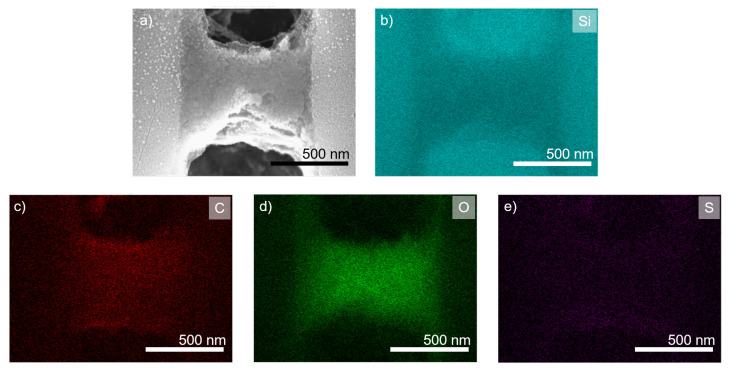
SEM image and EDX analysis of a cross sectional view of seven bilayers PDADMAC/PSS on isoporous silicon support with fully air-filled support during polyelectrolyte complexation. (**a**) SEM image, (**b**) EDX silicon signal, (**c**) EDX carbon signal, (**d**) EDX oxygen signal, and (**e**) EDX sulphur signal.

**Figure 8:**
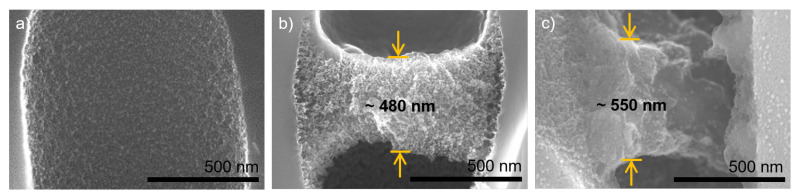
FESEM imaging of porous silicon support cross-sections. (**a**) no coating; (**b**) three bilayers PDADMAC/PSS with air-filled support during polyelectrolyte complexation (EDX can be found in Supplementary Materials Figure S1); (**c**) seven bilayers PDADMAC/PSS with air-filled support during polyelectrolyte complexation.

The formed polyelectrolyte complex structures were prepared in liquid nitrogen and optically analyzed by field-emission scanning electron microscopy (FESEM) with either a Hitachi S4800 (Figure 2, Figure 3, Figure 4 and Figure 5), or a Hitachi SU9000 with an Oxford Instruments EDX module (Figure 6, Figure 7 and Figure 8). All samples for scanning electron microscopy were lyophilized for sample preparation.

Layer growth was measured for reference on solid silicon wafers coated with PEMM with the same procedure as the isoporous silicon support structures. Polyelectrolyte multilayer (PEM) thickness was investigated with an RC2 dual rotating compensator variable angle spectroscopic ellipsometer (J.A. Woollam, Co., Lincoln, NE, USA). Two sets of experiments were performed: air-dried samples and samples stored in water.

The brittle nature of the isoporous silicon support structures prevented conventional retention measurements of the formed polyelectrolyte complex structures. The supports did not withstand the normal filtration conditions of several bars transmembrane pressure. Therefore, we designed a test cell for diffusion dialysis characterization based on Xu et al. [46]. The produced PEMM with the isoporous support structures were conditioned in a 0.05 M electrolyte solution overnight. The structures were then assembled between two sealed compartments, each with approximately 40 mL volume. These compartments were each equipped with a pH and a conductivity sensor. As the PEMM was located closer to one pore end than to the other, the compartment closer to the PEMM structure was filled with 0.1 M electrolyte solution with NaCl, Na_2_SO_4_, MgCl_2_, and MgSO_4_, respectively. The other compartment was filled with distilled water. Constant stirring of the liquids in both compartments avoided concentration polarization. This diffusion dialysis endured for 3 h. Liquid volumes in the compartments were monitored over time. Additionally, conductivity and pH before and after the diffusion dialysis were logged. The ionic flux was calculated according to Equation (2) with *n* being the amount of ions transferred in total, *t* being the time of diffusion dialysis, and $A_{Membrane}$ being the available membrane area.

(2) $$I_{salt}=\frac{n}{A_{Membrane}\cdot t\cdot \Delta c}$$

To account for the driving forces changing non-linearily during the diffusion analysis, the mean logarithmic concentration difference Δc is used for the determination of the ionic flux according to Equation (3). For reference, a commercial nanofiltration membrane (NF270, FilmTec) was characterized in diffusion dialysis behavior.

(3) $$\Delta c=\frac{\left(c_{0}^{f}-c_{0}^{p}\right)-\left(c_{t}^{f}-c_{t}^{p}\right)}{ln\frac{c_{0}^{f}-c_{0}^{p}}{c_{t}^{f}-c_{t}^{p}}}$$

For the manufactured hollow fiber modules, the pure water permeance in L/(m^2^ h bar) was measured in dead-end module configuration. 1 bar transmembrane pressure (TMP) was applied using a pressure vessel. Permeance was determined for three different modules. Salt retention was measured in cross-flow filtration experiments for each salt individually. Salt concentrations of 0.1 M were used in the feed stream. The conductivity of retentate ($\sigma_{R}$) and permeate ($\sigma_{P}$) were measured using a conductivity meter (SevenCompact pH/Cond S213, Mettler Toledo, Columbus, OH, USA). Salt retention was calculated according to Equation (4).

(4) $$R_{salt}=100\%\cdot \left(1-\frac{\sigma_{P}}{\sigma_{R}}\right)$$

## 3. Results and Discussion

### 3.1. Polyelectrolyte Pore Bridging

In a first step, the effect of non-water-wetted pores during dip-coating was investigated by controlling the wetting state in the support via air back-pressure. In Figure 2, the results for polyelectrolyte complex formation depending on the pre-wetting agent are displayed. Figure 2a,c show the isoporous silicon support structure without any coating in top view (a) and cross-section (c). In Figure 2b,e, results are displayed for a water-pre-wetted support structure and coated with three bilayers of polyelectrolytes, meaning six coating steps. Polyelectrolytes fully covered the support structure. The polyelectrolytes entirely intruded the pores and covered the pore walls.

In contrast, a pore bridging layer can be seen in Figure 2c,f. Here, the support was filled with air during the dip-coating procedure of three bilayers and intrusion of the coating solutions was prevented by applying a minimum of excess pressure. This wetting control caused a polyelectrolyte complexation at the gas–liquid interface instead of a pore wall coverage. We presume that a thin film of polyelectrolyte-rich solution remained after the first coating and rinsing procedure, which allowed a complexation with the respective counter-polyion when dipped in the next coating bath. The obtained results were in good agreement with Mallwitz et al. [42]. Some polyelectrolyte pore bridges were ruptured. These ruptures can be reasoned with a too fast drying procedure during sample preparation for FESEM [42].

In contrast to other studies where a polyelectrolyte complex film is produced on top of the support [30], the evolved polyelectrolyte bridge was located some hundred nanometers away from the pore mouth. This indentation was caused by capillary forces pulling the liquid inside the pores during the coating process. The indented positioning of the active separation layer might be advantageous regarding industrial nanofiltration applications since a ceramic structure might reduce erosion of the more delicate polyelectrolyte structure underneath. However, this hypothesis needs further investigation in future studies.

A more detailed view of a cross-section with three bilayers PDADMAC/PSS is shown in Figure 6. All pores contained the polyelectrolyte pore bridges at the same position inside the pores. This uniformity indicates a successfully controlled polyelectrolyte complexation via interface design. The magnification in Figure 6b reveals that the wetting control successfully prevented polyelectrolyte intrusion inside the support. The arrows indicate the depth in the pore up to which polyelectrolytes covered the silicon surface.

Further evaluation of the polyelectrolyte bridge structure was conducted using energy-dispersive X-ray spectroscopy (EDX) measurements. For samples with three bilayers of PDADMAC/PSS, signals for silicon, carbon, and oxygen were found (see Figure S2). For seven bilayers of PDADMAC/PSS, results are displayed in Figure 7. Here, an additional sulfur signal strong enough for automatic detection was found that can be accounted to the contained PSS. The detected silicon signal can be attributed to the support structure, whereas the carbon signal can clearly be attributed to both of the polyelectrolytes. Oxygen can in general be found in both, the native oxide layer of the support structure, and the polyanion PSS. The native oxide layer on the support, however, was very thin and only causes some background signal, while the oxygen signal is much more prominent in the area of the polyelectrolyte complex. Hence, we concluded that this signal could be used for a localisation of PSS. Additionally, the sulphur signal was attributed to the contained PSS in the polyelectrolyte structure due to its chemical composition.

No layering structure could be observed within the polyelectroylte complexes. However, the carbon and the sulphur signal tended to show increased signal strength at the upper and lower edges of the complex structures. This can be reasoned with local de-swelling effects and local porous matrix collapse that increased the polyelectrolyte density in those areas. Overall, all signals showed an even distribution within the polyelectrolyte pore bridging structure that might indicate intermixing and co-adsorption effects during the manufacturing process. However, no definite conclusion about the mechanisms during the wetting-controlled complexation could be drawn from the results obtained in EDX.

### 3.2. Polyelectrolyte Multilayer Growth

Layer growth of the polyelectrolyte multilayer has been intensively studied in the literature [47,48,49,50]. For dip-coating processes, different mechanisms causing subsequent linear, exponential, and again linear layer growth are known from literature [47]. Further, nanoconfinement on one side of the PEMM affects the polyelectrolyte structure significantly [51]. Hence, nanoconfinement at the pore walls and the lack of influence of support beneath the polyelectrolyte structure might affect the layer growth of pore bridging structures.

Figure 8 shows cross-sections of single pores with no coating (a), three bilayers (b), and seven bilayers (c), respectively. In the coated samples, air was used to prevent intrusion of the coating solution. The thickness of the three bilayer structure is determined to approximately 480 nm at the center of the spanned support pore. With more than twice the number of coating steps, a thickness increase to only 550 nm could be observed for the seven bilayer structure. This indicated a decrease of layer thickness growth after the first coating steps.

Additionally, the internal structure of the two polyelectrolyte complex structures distinctly differed from each other. While the three bilayer structure showed a open porous matrix, the seven bilayer structure appeared rather dense. The latter might be reasoned with a collapse of the polyelectrolyte complex formation, as the confinement of the pore walls was no longer symmetrically present due to the rupture in the polyelectrolyte structure at the right side of the pore. This is further discussed below after comparing the observed polyelectrolyte complex thicknesses to conventionally dip-coated structures.

**Figure 9:**
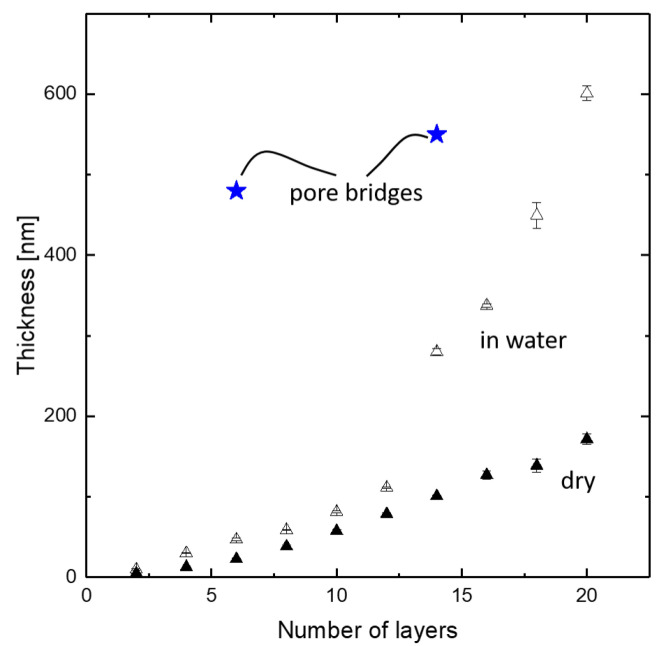
PDADMAC/PSS layer thickness on silicon wafers in function of number of coating steps measured by ellipsometry (triangles) in aqueous environment and air-dried as well as pore-bridging layer thicknesses taken from Figure 8.

For reference to the PEMM thickness evaluation, we dip-coated a silicon wafer with a native oxide surface with identical coating procedures as the isoporous silicon structures. Afterward, polyelectrolyte layer thicknesses were analyzed by ellipsometry, both in a wet and in a dry state. Three different positions at each sample were measured in thickness. Results are displayed in Figure 9. Thicknesses of the polyelectrolyte pore bridge structures taken from Figure 8 are indicated for reference.

The effect of swelling in water can be seen in the data obtained by ellipsometry. The pore bridging structures showed a thickness of almost one order of magnitude higher than the PEMM on a plain silicon wafer. The increase from three bilayers (saying six coated layers) to seven bilayers (saying 14 coated layers) was in the same range as the increase in the thickness of the reference samples obtained with a flat silicon wafer. The fact that there was no solid support but a second fluid phase underneath the formed polyelectrolyte complex structure might have effected the dominating mechanisms in several ways.

First, we expected a significant amount of residual polycations close to the fluid–fluid interface even after the first rinsing step due to the surfactant nature of polyelectrolytes a possible diffusion limitation during rinsing. These residual polycations then co-adsorbed during the coating with polyanions. This could lead to significantly thicker polyelectrolyte complex layers than conventional dip-coating.

Second, the missing nanoconfinement at the fluid–fluid interface can alter the diffusion limitation causing linear growth in the first bilayers of conventional dip-coating [47]. Hence, further layer growth is less hindered. This, in turn, can lead to faster exponential or linear growth even at a low number of applied layers.

Third, the support might have an additional influence on the prevalent driving forces. With the support pore walls in orthogonal orientation to the formed polyelectrolyte structure, the support delivers additional surface area for nanoconfinement (see Figure 6). Combined with the fluid–fluid interface, this leads to a large adsorption surface to volume ratio in the support capillaries. This, in turn, leads to higher electrostatic interactions and, hence, to more adsorbed polyelectrolytes. Additionally, if not a fully bridging structure is formed during the first bilayer of polyelectrolyte adsorption, a radial growth of the polyelectrolyte structure is to be expected from the support pore walls towards the pore center. In this case, the support stabilizes the formed polyelectrolyte complex and prevents the complexes from being flushed away from the support.

An additional factor biasing the layer thickness results displayed in Figure 9 is the different shrinking behavior by polyelectrolyte deswelling inside the pores due to the nanoconfinement of the pore walls. In Figure 8b,c, the textures of the polyelectrolyte complex structures appear different. Furthermore, the structure in Figure 8c is ruptured, whereas the structure consisting of three bilayers appears to be intact. This difference in shrinking behavior might have a significant influence on the visible layer thickness in FESEM imaging. An exemplary visualization is given in Figure 3 on two neighbouring pores.

Figure 3 shows a cross-section of an isoporous silicon support structure with three bilayers of PDADMAC/PSS coating using air-liquid interface control during polyelectrolyte complexation. Here, directly neighboured pores show distinctly different polyelectrolyte pore bridging structures. First, the layer thickness increases three-fold. Second, the texture appears dense for the flat structure, whereas it appears as an open porous complex for the thicker structure. It can be noted that the thicker complex is partially detached from the pore wall. This can be reasoned with stresses on the polyelectrolyte complexes by nanoconfinement at the pore walls.

During the drying procedure, mechanical stresses due to polymer shrinking arise. The different appearance of polyelectrolyte structures in Figure 3 indicate that these stresses lead to shrinking along the pore axis if the nanoconfinement at the walls remains intact. In contrast, these stresses lead to a predominantly radial shrinking of the polyelectrolyte structure when the polymer partially detaches from the pore wall. In turn, this means that the thicknesses that are taken from Figure 8, especially Figure 8c can only be taken as rough values, as their shrinking behavior during drying needs further investigation in future studies.

### 3.3. Separation Behavior

Nanofiltration membranes are conventionally characterized by molecular weight cut-off, free water flux, and ion rejection. The material system of PDADMAC/PSS PEMM is well known, and characteristics are reported in the literature [52,53,54]. For the high amount of counter ions used for polyelectrolyte layer fabrication, however, high polyelectrolyte adsorption rates but low ion retention are reported [44,45].

For all three typically characteristic parameters, the membrane must be pressurized. Since the isoporous support presents its brittle nature by tending to break even at low shear stresses, a classical characterization of the formed polyelectrolyte complex structures was not feasible. Instead, a diffusion dialysis module was built to investigate the formed polyelectrolyte complex structures as diffusion barriers [35,46]. However, measuring these properties requires pressurizing the membrane. As the isoporous silicon support structures are very brittle and tend to break even at low shear stresses, it was not possible to measure the properties mentioned above. Instead, a diffusion dialysis module was built to investigate the formed polyelectrolyte structures as diffusion barriers.

This way, the polyelectrolyte pore bridging structures were investigated regarding their diffusive behavior of NaCl, Na_2_SO_4_, MgCl_2_, and MgSO_4_ in single salt experiments. For each salt, a non-coated, fully water-wetted isoporous support structure and support containing pore bridging polyelectrolyte complexes of three bilayers PDADMAC/PSS was investigated.

**Figure 10:**
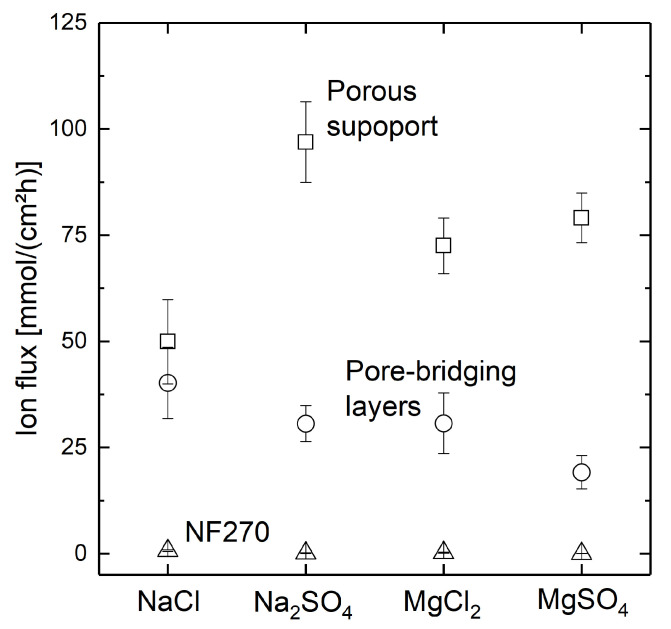
Ion Flux of NaCl, Na_2_SO_4_, MgCl_2_, and MgSO_4_ across a porous support structure with three bilayers of PDADMAC/PSS coated with a fully water-wetted support, a porous support structure coated with three bilayers of PDADMAC/PSS with air-filled pores during coating, and a commercial NF270 nanofiltration membrane for reference. Experiments were performed with three membranes each.

The ion flux resulting across the membrane is displayed in Figure 10 for the porous support with three bilayers of PDADMAC/PSS with a fully water-wetted support (conventional dip-coating), the porous support containing pore-bridging polyelectrolyte complexes after three bilayers of PDADMAC/PSS-coating, and a commercial NF270 nanofiltration membrane for reference.

For the conventionally dip-coated support, highest ion flux is observed. Considering the residual pore sizes observed displayed in Figure 8, no ion retention was anticipated for this wetting configuration. Due to the intrinsic low ion rejection of the polyelectrolyte system at the chosen counter ion strength in the coating solution, the formed polyelectrolyte layers could not compete with commercial nanofiltration membranes. However, a salt-dependent change in ion flux can be observed when comparing the ion fluxes through the non-coated supports to the polyelectrolyte-coated ones. As the order of ion deflections is shifted due to the coating as expected from literature [55,56], this is a strong indication for a polyelectrolyte-dominated diffusion dialysis behaviour and, hence, a successful formation of a pore-covering polyelectrolyte coating. Yet, different coating compositions are necessary to obtain ion-selective polyelecrolyte layers that show ion rejections desirable for nanofiltration applications.

### 3.4. Miscible and Immiscible Wetting Agents

In the results presented above, conventional dip-coating with fully water-wetted supports was compared to air-filled pores of the isoporous silicon support structure during dip-coating. The results for three bilayers of PDADMAC/PSS with these two wetting agents are displayed in Figure 4a,b for reference. In Figure 4c, also a cross-section of air-filled support during dip-coating is displayed. However, wetting control was disabled after the first two coating steps in this case to test the stability of the so far complexes. Afterward, two more bilayers were dip-coated on the sample. Still, most pores contained pore-bridging polyelectrolyte structures. The formed structures, however, are significantly thinner than the samples with constant wetting control. In Figure S2, a top view of this sample is given. It can be seen that pore bridging structures formed in nearly all pores. However, none of these structures fully spanned over the support pores. The structures formed showed round defects in most cases. This is a further indication for a radial growth of the formed polyelectrolyte complexes, as hypothesized above.

As a water-miscible wetting agent, PEG400 was inserted into the pores before dip-coating. Three bilayers of PDADMAC/PSS were coated on the PEG400-filled support. The result is shown in Figure 4d. As with air-filled pores, pore-bridging polyelectrolyte structures are formed some hundred nanometers along the pore throat. After coating, residual PEG400 could easily be removed by storing the sample in water over 48 h.

We concluded that two factors presumably influenced the nature of the polyelectrolyte architectures after few coating steps. First, mainly capillary effects determined the location of the pre-wetting/coating solution phase boundary. This location definesd the position of the formed polyelectrolyte complexes. Second, the extent of diffusion limitation of the remaining polyelectrolytes close to the phase boundary was decisive on how much polyelectrolyte material was co-adsorbed during the subsequent coating step.

### 3.5. Heteroporous Support Structures

For a general proof of concept of pore-spanning polyelectrolyte complex structures on heterogeneous supports, we adapted the wetting control to a commercial hollow fiber membrane. For visual evaluation of both the wetting control and the polyelectrolyte structure localization, a pore size of approximately 1 μm was desired. This pore size was more than one order of magnitude higher than conventional supports for nanofiltration membranes. A Pentair X-Flow membrane, which is a polymeric PES/PVP ultrafiltration membrane with the selective layer on the lumen side, was chosen as model support. The shell side of these membranes had a wide-open porous structure, as shown in Figure 5a. This shell-side of the support membrane was coated with polyelectrolytes to form a selective layer. A constant back-pressure of air applied to the lumen channel was used to prevent coating solution from intruding the pores of the support during dip-coating. Five bilayers of PDADMAC/PSS were applied. A cross-section of a coated heteroporous support structure is given in Figure 5b.

After five bilayers of polyelectrolyte coating, no pore intrusion by polyelectrolytes could be observed, as shown in Figure 5b. In contrast to the coated flat isoporous structures, the polyelectrolyte complexes were located at the support structure’s outer surface. This positioning can be explained with the adapted wetting control that caused a minimal constant over-pressure of the gas phase.

The pure water flux of the non-coated membrane was determined to be approximately 250 LMHbar. The available membrane was calculated on the membrane shell, whereas the ultrafiltration layer was located at the lumen of the support fiber. After applying five bilayers of polyelectrolyte coating on the shell side of the support fiber, the flux was reduced to 60 ± 15 LMH. The significant reduction in flux after polyelectrolyte coating suggested an at least partially successful coating of the porous support. However, the still high pure water permeabilities also indicated remaining defects in the polyelectrolyte coating. Applying TMPs of 1.5 bar or higher led to a drastic increase in permeability, implying the rupture of applied polyelectrolyte coating.

The separation behavior of manufactured hollow fiber membranes was tested for MgCl_2_ and MgSO_4_ at 1 bar TMP with 0.1 M feed solution concentrations in outside-in permeation. The observed retentions are displayed in Figure 5c. As previously discussed, the maximum achievable ion rejection with the chosen coating solution composition was fairly low [44,45]. Hence, even the small retentions observed are an indication for a partially successful pore coverage with polyelectrolytes. Yet, the retentions remained below the ones reported in the literature for the used material system, indicating defects in the manufactured polyelectrolyte layer.

We conclude that more coating steps are necessary to obtain a full support coverage with the used material system. However, the partial pore coverage even at a number of five bilayers on a porous support with pores in the micrometer range is a promising result, since even on an isoporous support pores of this size could not be covered with conventional dip-coating. Future work will be necessary to find an optimum between ion selectivity, mechanical stability, and tolerable support pore size.

## 4. Conclusions

A novel manufacturing method for ion-selective polyelectrolyte multilayer membranes on or inside porous support structures is presented. This method uses support wetting control during dip-coating, which allows interfacial polyelectrolyte deposition at a gas–liquid or liquid–liquid phase boundary inside the porous support structure. This way, pores as large as 1 μm can be spanned with polyelectrolytes with few coating steps. Polyelectrolyte depositions are located via FESEM analysis and evaluated in their composition using EDX. Polyelectrolyte coating thickness can be tuned by varying the number of coating steps. Additionally, using PEG400 as a miscible pre-wetting agent leads to a decrease in polyelectrolyte deposition thickness. Further, layer positions can be adjusted by tailoring the fluid–fluid interface location during dip-coating.

The polyelectrolyte complex structures produced in this study as a proof of concept are shown to be an ion-selective diffusion barrier. Adapting the method to hollow fiber heteroporous systems with pore sizes in the micrometer range a partial coverage of the support can be achieved by applying only five bilayers of polyelectrolyte coating. Both the resulting pure water flux and the low retention of salts indicate remaining defects. Yet, the formation of a ion-retaining polyelectrolyte layer within few coating steps is promising for further optimization of this method. To make this method applicable for larger-scale membranes, different coating compositions need to be tested to enhance the achievable ion selectivity of the formed polyelectrolyte complexes. Further, the trade of between needed mechanical stability for nanofiltration applications and tolerable maximum pores size will be evaluated in future studies.

## Data Availability

The data that support the findings of this study are available from the corresponding author upon reasonable request.

## Supplementary Materials

The following are available online at https://www.mdpi.com/article/10.3390/membranes11090671/s1, Figure S1: Three bilayer SEM and EDX analysis. Figure S2: Fractured polyelectrolyte bridges from intermittent wetting control.

## Abbreviations

The following abbreviations are used in this manuscript:

*A*	Effective membrane area
*d*	pore diameter
Δc	logarithmic mean concentration difference
EDX	Energy Dispersive X-ray spectroscopy
FESEM	Field Emission Scanning Electron Microscopy
I	ionic flux
LbL	Layer by Layer
LMH	L/(m_2_ h)
MDPI	Multidisciplinary Digital Publishing Institute
*n*	number of ions transported
PAA	Polyacrylic Acid
PAH	Poly(allylamine hydrochloride)
*p_b_*	breakthrough pressure
PDADMAC	Poly(diallyldimethylammonium chloride)
PEG400	Polyethylene Glycol 400 Da
PEM	Polyelectrolyte Multilayer
PEMM	Polyelectrolyte Multilayer Membrane
PES	Polyethersulphone
PSS	Poly(styrenesulfonate)
PVP	Polyvinylpyrrolidone
*R*	Retention
*t*	Time of Diffusion Dialysis
TMP	transmembrane pressure
