# Supplementary material for: Wetting-Induced Polyelectrolyte Pore Bridging
Source: Membranes (Basel). 2021 Aug 31;11(9):671. doi: 10.3390/membranes11090671 (PMC8466633; doi:10.3390/membranes11090671)
Supplement: Supplementary file 1 [file membranes-11-00671-s001.zip › membranes-1341506-supplementary.pdf]

Supplementary Materials: Wetting-induced Polyelectrolyte Pore Bridging

Anna Kalde [1], Johannes Kamp [2], Elizaveta Evdochenko [2], John Linkhorst [2], and Matthias Wessling [1,2,*]

1. S1

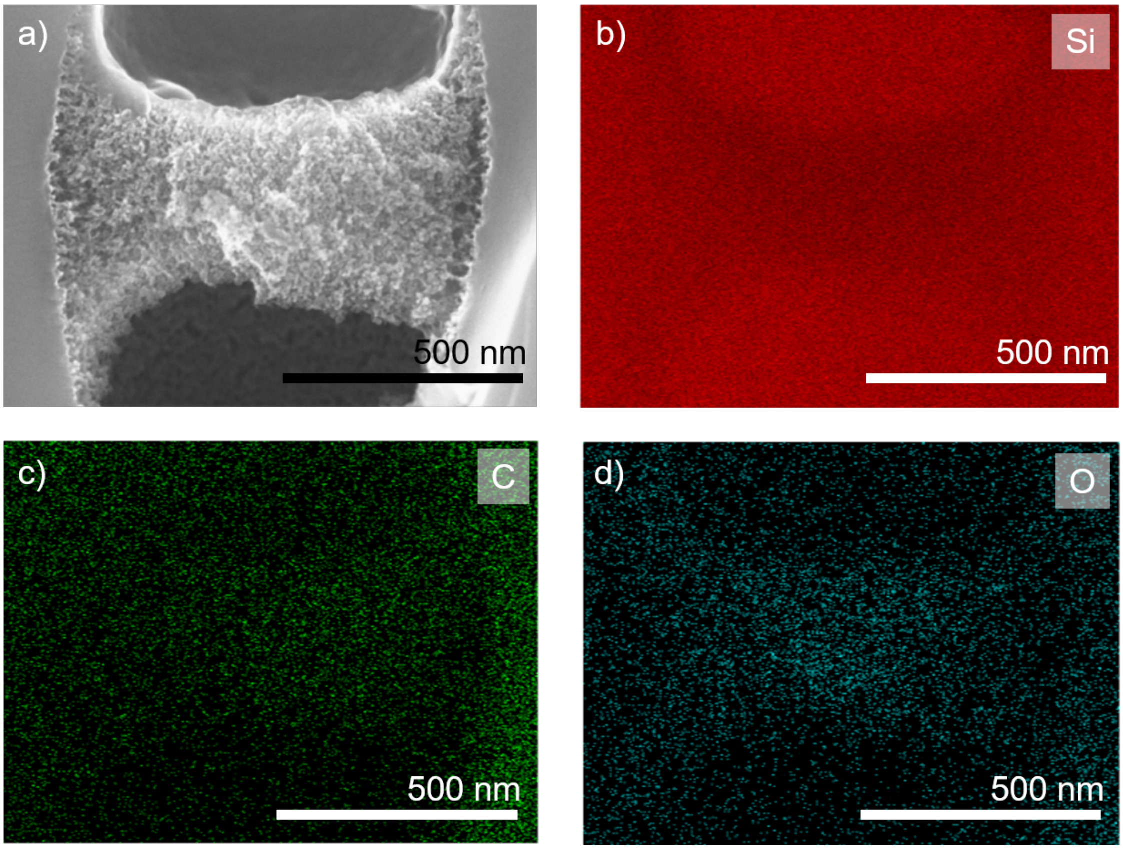

Figure S1. SEM image and EDX analysis of a cross sectional view of three bilayers PDADMAC/PSS on isoporous silicon support with fully air-filled support during polyelectrolyte complexation. a) SEM image, b) EDX silicon signal, c) EDX carbon signal, d) EDX oxygen signal.

2. S2

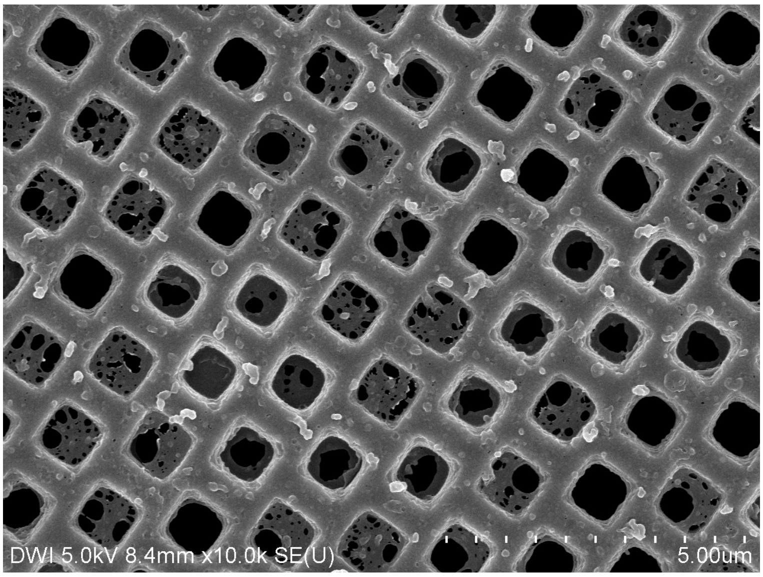

Figure S2. FESEM imaging of isoporous silicon support structures. Top view of three bilayers PDADMAC/PSS on silicon support with air-filled support during the first two coating steps and no wetting control for further coating.
